# Inequalities in healthy life expectancy by Brazilian geographic regions: findings from the National Health Survey, 2013

**DOI:** 10.1186/s12939-016-0432-7

**Published:** 2016-11-17

**Authors:** Célia Landmann Szwarcwald, Paulo Roberto Borges de Souza Júnior, Aline Pinto Marques, Wanessa da Silva de Almeida, Dalia Elena Romero Montilla

**Affiliations:** Institute of Communication and Information Science and Technology in Health, Oswaldo Cruz Foundation, Rio de Janeiro, Brazil

**Keywords:** Healthy life expectancy, Inequalities, Geographic regions, National health survey, Brazil

## Abstract

**Background:**

The demographic shift and epidemiologic transition in Brazil have drawn attention to ways of measuring population health that complement studies of mortality. In this paper, we investigate regional differences in healthy life expectancy based on information from the National Health Survey (PNS), 2013.

**Methods:**

In the survey, a three-stage cluster sampling (census tracts, households and individuals) with stratification of the primary sampling units and random selection in all stages was used to select 60,202 Brazilian adults (18 years and over). Healthy life expectancies (HLE) were estimated by Sullivan’s method according to sex, age and geographic region, using poor self-rated health for defining unhealthy status. Logistic regression models were used to investigate socioeconomic and regional inequalities in poor self-rated health, after controlling by sex and age.

**Results:**

Wide disparities by geographic region were found with the worst indicators in the North and Northeast regions, whether considering educational attainment, material deprivation, or health care utilization. Life expectancy at birth for women and men living in the richest regions was 5 years longer than for those living in the less wealthy regions. Modeling the variation across regions for poor self-rated health, statistically significant effects (*p* < 0.001) were found for the North and Northeast when compared to the Southeast, even after controlling for age, sex, diagnosis of at least one non-communicable chronic disease, and schooling or socioeconomic class. Marked regional inequalities in HLE were found, with the loss of healthy life much higher among residents of the poorest regions, especially among the elderly.

**Conclusions:**

By combining data on self-rated health status and mortality in a single indicator, Healthy Life Expectancy, this study demonstrated the excess burden of poor health experienced by populations in the less wealthy regions of Brazil. To mitigate the effects of social exclusion, the development of strategies at the regional level is essential to provide health care to all persons in need, reduce risk exposures, support prevention policies for adoption of healthy behaviors. Such strategies should prioritize population groups that will experience the greatest impact from such interventions.

## Background

Brazil is a middle-income country with large socioeconomic inequalities and extreme disparities in the distribution of income. These social inequalities may affect people’s health status directly [1, 2] and also indirectly, by limiting their access to and utilization of health services [3–6]. Health inequalities found nationwide may therefore be considered a product of the poor living conditions experienced by a considerable fraction of the Brazilian population [7, 8].

Brazil is politically and geographically divided into five distinct regions (North, Northeast, Southeast, South and Center-West) with varied physical, demographic, and socioeconomic characteristics. Among the five Brazilian geographic regions, the North and the Northeast are the most deprived. The Southeast has greater economic importance and includes more than 40 % of the Brazilian population, including the most populous states of São Paulo and Rio de Janeiro. The South has the smallest territorial area, comprises 14 % of the total population, and is one of the two wealthiest regions. The Center-West has an intermediate stage of development that has accelerated since Brazil’s capital was moved to Brasilia in the 1960s. The concentration of poor socioeconomic conditions in the North and Northeast regions and inequalities in the distribution of health services and resources are reflected in a steep North-South gradient in many health indicators [9].

In Brazil, highlighting inequalities at the regional level proved to be especially important to promote actions and programs to decrease socioeconomic gaps. In the late 1990’s, the Family Health Program was implemented as a national policy for primary care, giving priority to municipalities with the worst socioeconomic levels, especially those located in the North and Northeast [10]. The expansion of primary care in deprived areas showed important impacts: significant shrinkage in the historic regional gap in infant mortality rates [11]; important decline in unnecessary hospitalizations [12]; decrease in under-five mortality rates due to ill-defined causes and unattended deaths [13]; and reduction of mortality from heart and cerebrovascular diseases through cardiovascular disease prevention, care and follow-up [14].

Historically, national studies have focused on regional health inequalities emphasizing differences in mortality [15, 16]. The demographic shift and the epidemiologic transition in Brazil have, however, drawn attention to other ways of measuring population health [17, 18]. The growth in longevity has been accompanied by an increase in disability and in non-communicable chronic diseases that are changing the morbidity-mortality profile [19].

In this new scenario, different health indicators have been proposed to complement mortality by additionally accounting for morbidity and quality of life [20]. In national health surveys, self-rated health and self-reported diagnosis of chronic non-communicable diseases have been broadly used to establish differences in morbidity among population groups [21–23]. Health indicators that combine mortality data with morbidity or health status data have also been proposed for evaluating health care and prevention programs by placing greater emphasis on the quality of life in later years [24, 25].

Among the distinct health indicators that incorporate morbidity and mortality in a single measure, healthy life expectancy (HLE) obtained by Sullivan’s method [26] has been the most frequently used due to its mathematical simplicity, the availability of required data, and the ease of interpreting results. This indicator is a population health measure that estimates the expected number of “healthy years” (years of life in good health) for persons at a given age. Definitions of “healthy” are usually based on self-rated health, long-term illness or disability, and functional or cognitive limitations [27].

In Brazil, healthy life expectancy has been estimated for the total adult population [25, 27, 28] but regional differences in healthy longevity have been less well studied. Given the evidence indicating that differences in living conditions affect the regional pattern of morbidity and mortality in the country [29], this paper investigates regional inequalities in healthy life expectancy based on information from the National Health Survey, 2013.

## Methods

As part of a project developed by the Ministry of Health (MoH) aimed at assessing health system performance of Brazilian states and regions, the National Health Survey (*Pesquisa Nacional de Saúde* - PNS) was carried out in Brazil in partnership with the Brazilian Institute of Geography and Statistics (IBGE). The project was approved by the National Commission of Ethics in Research (CONEP) in June 2013 (No. 328.159).

The IBGE was responsible for the PNS sampling and fieldwork and the MoH was responsible for the PNS contents and financing.

### Sampling

The National Health Survey (PNS) is a national household based survey. The surveyed population consisted of residents of private households in Brazil, excluding those located in special census tracts (military bases, lodges, camps, boats, prisons, asylums, orphanages, convents and hospitals). The sample used in the National Health Survey (PNS) is a subsample of the Master Sample of the Integrated Household Survey System (SIPD, IBGE), whose geographical scope comprises the census tracts of the Geographic Operating Base 2010 Population Census, except those with very small number of households and special sectors [30].

A three-stage cluster sampling (census tracts, households and individuals) was used with stratification of the primary sampling units (PSUs) and random selection in each stratum. Census tracts or set of sectors compose the primary sampling units (PSUs), households are the units of the second stage and residents aged 18 or older define the third-stage units. As part of the Integrated Household Survey System, the PSUs were obtained from the Master Sample, using the same PSU stratification.

In each PSU, from 10 to 14 households were selected using a simple random sample from the National Register of Addresses for Statistical Purposes (CNEFE, IBGE). In each selected household, an adult (18 years old and over) was selected with equal probability to answer the questionnaire through a face-to-face interview [30].

### Fieldwork

Interviewers, supervisors and coordinators of the IBGE carried out the PNS fieldwork. Training materials were prepared in partnership with the MoH. Coordinators and supervisors were trained in person and then they trained all field staff to conduct interviews using PDAs (Personal Digital Assistants) and to perform anthropometric and blood pressure measurements. A consortium of private laboratories carried out the collection and laboratory tests on the biological samples (blood and urine) [31].

The PNS fieldwork was carried out from August, 2013 to February, 2014 with 6,069 selected census tracts and 81,254 visited households. From these, 69,994 households were occupied, 64,348 household interviews were conducted, and 60,202 individual interviews completed. Response rates by state are available in a previous publication [32].

### Measurement instrument

The PNS questionnaire is divided into three parts: characteristics of the household (water and sanitation, electricity, household assets); demographic and health information on all household residents (sociodemographic characteristics, access and utilization of health care, private health insurance coverage for all household members); and the individual questionnaire, which includes modules on self-perception of health, accidents and violence, life styles, non-communicable chronic diseases, women’s health, oral health, health care use, and assessment of the quality of care received. The entire questionnaire is available at the PNS site (www.pns.fiocruz.br). Only one household member (key informant) answers the first and second parts for all household residents. The individual questionnaire is answered by a resident of 18 years and older, selected with equal probability among all adult household residents as described above.

### Main indicators

In this paper, to show socioeconomic differences by Brazil’s five macro-geographic regions, we used indicators of educational attainment, health care access and utilization, and coverage by a private health plan. We calculated an index based on the number of household assets, degree of education of the household head and presence of monthly paid housekeeper, using a standard Brazilian classification scheme for social class: (A/B) upper class; C (middle class); and lower class (D/E), adapting the approach of the Brazilian Association of Survey Firms [33].

The standard method for assessing social class in Brazil combines the household asset index, calculated by a sum of points attributed to the number of households goods with larger weights attributed to certain high-value items, and the level of instruction of the household head, with higher point attributed to greater educational attainment. The sum of household points is then aggregated in intervals to define social class categories: D/E (0–13 points); C (14–23 points); A/B (24–50 points).

Healthy life expectancies were estimated by Sullivan’s method [26] according to sex, age and geographic region. The approach is an adaptation of the traditional life table method using two independent measures of health: the specific rate of being healthy by age group and the mortality component given by age-specific life expectancy provided by the IBGE [34]. The method consists of removing the proportion of time lived in poor health from the total expected lifespan of a given cohort [27]:

$$ \mathrm{H}\mathrm{L}\mathrm{E}=\frac{1}{{\mathrm{l}}_{\mathrm{x}}}{\displaystyle \sum_{\mathrm{x}}^{\mathrm{w}}\Big(1\hbox{-} {}_{\mathrm{n}}}{\uppi}_{\mathrm{x}}\Big){}_{\mathrm{n}}\mathrm{L}_{\mathrm{x}} $$

where l_x_ is the number of survivors at the exact age x; _n_π_x_ represents the prevalence of a determined state of health (poor self-rated health) among individuals with ages in the interval (x, x + n); _n_L_x_ is the total number of years lived by a cohort in the age group (x, x + n); and w represents the largest age category.

To establish the “unhealthy state” we used self-rated health based on the following PNS question: “In general, how would you rate your health? “with five possible answers (very good, good, moderate, bad, very bad). The first three options were aggregated to define “good health” and the two last categories to define “poor self-rated health”. The age specific rate of being unhealthy was estimated by the proportion of people reporting poor self-rated health in each 5-year age group.

### Data analysis

Logistic regression models were used to investigate socioeconomic and regional inequalities in poor self-rated health, after controlling by sex and age. The effects of geographic regions (considered as dummy variables) were also adjusted by a binary variable representing diagnosis of at least one non-communicable chronic disease.

As the PNS design used stratification of census tracts and multiple stage cluster selection, the complex sample design was considered in the statistical analysis.

## Results

### Inequalities by geographic regions

Table 1 shows the distribution of people aged 20 years and over by geographic region according to socioeconomic characteristics. Wide disparities were found by region with the worst indicators consistently found in the North and Northeast. In relation to educational attainment, while in the Southeast over 50 % completed secondary school, in the Northeast, this proportion is lower than 40 %. In the Northeast, more than one fifth of adults is illiterate. As to socioeconomic class, in the North, more than 40 % of the adult population is classified in the lowest classes (D/E) and less than 20 % in the upper classes A and B. In contrast, in the South and Southeast, 40 % or more belongs to class A/B and less than 20 % in class D/E. Coverage of private health plans varied from 12.6 % (North) to 34.5 % (Southeast). Differences in the distributions by macro geographic regions were all statistically significant (*p* < 0.001).

**Table 1:** Socioeconomic and health indicators among adults aged 20 years and over^c^ by Brazilian regions, 2013

Indicators	Geographic Region					Brazil	*p*-value^*^
	North	Northeast	Southeast	South	Center-West		
Schooling							*p* < 0.001
Illiterate	19.4	23.0	9.9	9.7	12.4	14.2	
Incomplete elementary school	23.4	26.4	24.8	29.3	25.3	25.8	
Incomplete secondary school	15.5	12.9	14.4	15.5	14.9	14.3	
Complete secondary school	41.7	37.7	50.9	45.5	47.4	45.7	
Socioeconomic class							*p* < 0.001
A/B	16.8	18.6	40.2	42.8	34.0	32.7	
C	42.3	38.9	44.7	43.6	44.5	42.8	
D/E	40.9	42.5	15.1	13.6	21.5	24.5	
% Adults living in rural areas	21.7	23.8	6.9	15.0	8.9	13.8	*p* < 0.001
% Adults who consulted a doctor in the last 12 months	66.4	69.7	79.5	78.6	73.8	75.4	*p* < 0.001
Coverage (%) of private health plan	12.6	14.7	34.5	31.4	28.0	26.7	*p* < 0.001
Diagnosis of at least one NCD	39.0	44.4	47.4	53.2	44.8	46.7	*p* < 0.001
Life Expectancy at birth (years)^a^							-
M	68.2	68.1	73.3	73.5	71.2	71.3	
F	75.3	76.5	79.5	80.3	77.9	78.1	
T	71.5	72.2	76.6	76.9	74.4	74.8	
Number of doctors per 1000 population^b^	0.9	1.09	2.51	2.06	1.76	1.86	-
Poor self-rated health							
M	5.8	7.0	3.5	4.0	5.0	4.8	*p* < 0.001
F	7.6	9.4	5.2	6.5	6.0	6.7	*p* < 0.001
T	6.8	8.3	4.4	5.3	5.5	5.8	*p* < 0.001

Sources: National Health Survey, 2013; ^a^Brazilian Institute of Geography and Statistics, IBGE; ^b^Ministério da Saúde, Datasus, Indicadores e Dados Básicos

^c^Weighted Sample size 57,287

^*^ *p*-value of the chi-square test for comparison between geographic region distributions

Differences in health care utilization are also pronounced. The proportion of adults who consulted a doctor in the last 12 months ranged from 66.4 % in the North to 79.5 % in the Southeast. Great inequalities in the indicator of health resource supply are also found: in the Southeast, the number of doctors per 1000 inhabitants is almost three times higher than in the North. Barriers to access for diagnosis of long-term illness result in unequal prevalence rates, with the lowest proportion (39 %) of diagnoses of at least one non-communicable chronic disease found in the North (Table 1).

Life expectancy by geographic region also varied directly with socioeconomic development: the wealthier the region, the greater the longevity. The North and the Northeast have the shortest life expectancy at birth for both men and women. Life expectancy at birth of women and men living in the richest regions was 5 years longer than that of those living in the less developed regions (Table 1).

The proportion of adults with poor self-rated health by sex and geographic region is also shown in Table 1. Marked regional inequalities were found with the richest regions having the smallest proportions of poor self-rated health for both sexes. The proportion of poor self-rated health in Brazil was 5.8 % and ranged from 4.4 % in the Southeast to 8.3 % in the Northeast. Comparing the proportions by sex, females have greater proportions of poor self-rated health in all regions.

### Inequalities in self-rated health

Results of the logistic regression model of poor self-rated heath are presented in Table 2. The odds-ratio (OR) for females is 1.41 (*p* < 0.001) when compared to males and increases with age. The chance of having poor self-rated health is nine times higher for persons aged 70 years and over as compared to young adults aged 20 to 29 years. Statistically significant effects (*p* < 0.001) were found for all four regions when compared to the Southeast, with higher effects corresponding to the Northeast and to the North (Table 2).

**Table 2:** Results of logistic regression models having poor self-rated health as the response variable. Brazil, 2013

Variables		OR	95 % CI	*p*-value
Sex	F	1.41	1.26–1.58	*p* < 0.001
	M	1.00	-	-
Age group	20–29	1.00	-	-
	30–39	1.80	1.36–2.37	*p* < 0.001
	40–49	3.64	2.85–4.64	*p* < 0.001
	50–59	5.54	4.37–7.02	*p* < 0.001
	60–69	6.99	5.49–8.92	*p* < 0.001
	70+	9.21	7.15–11.87	*p* < 0.001
Region	North	2.01	1.64–2.48	*p* < 0.001
	Northeast	2.18	1.87–2.55	*p* < 0.001
	Southeast	1.00	-	-
	South	1.26	1.04–1.54	0.019
	Center-West	1.34	1.11–1.61	0.002

Modeling the variation among regions for poor self-rated health after controlling for educational level, age group, and sex (Table 3 Model 1A), the effects of the North and Northeast remain statistically significant (*p* < 0.001), the effect of the Center-West decreases but is still significant (*p* < 0.02), but the effect corresponding to the South is no longer statistically significant. After including the dummy variable representing diagnosis of at least one non-communicable chronic disease in the previous model (Model 2A), the largest effect corresponded to this variable, but no changes in the significance of the region effects were found. When we replace schooling with socioeconomic class in the two logistic regression models, we observe a more pronounced effect of household assets than educational attainment (models 2A and 2B). However, the effects of the North and Northeast persist (*p* < 0.001) when compared to the Southeast in both models.

**Table 3:** Logistic regression results: geographic region effects on poor self-rated health. Brazil, 2013

		Model 1A			Model 1B			Model 2A			Model 2B		
Variables		OR^a^	95 % CI	*p*-value	OR^b^	95 % CI	*p*-value	OR^c^	95 % CI	*p*-value	OR^d^	95 % CI	*p*-value
Sex	F	1.47	1.31–1.65	0.000	1.42	1.26–1.59	0.000	1.28	1.14–1.44	0.000	1.24	1.10–1.40	*p* < 0.001
	M	1.00	-	-	1.00	-	-	1.00	-	-	1.00	-	-
Age group	20–29	1.00	-	-	1.00	-	-	1.00	-	-	1.00	-	-
	30–39	1.54	1.17–2.02	0.002	1.93	1.46–2.55	0.000	1.26	0.95–1.67	0.113	1.60	1.20–2.13	*p* < 0.001
	40–49	2.62	2.05–3.35	0.000	3.84	3.01–4.91	0.000	1.74	1.36–2.23	0.000	2.56	1.99–3.28	*p* < 0.001
	50–59	3.58	2.83–4.54	0.000	5.57	4.39–7.06	0.000	2.00	1.56–2.56	0.000	3.08	2.39–3.96	*p* < 0.001
	60–69	3.91	3.05–5.01	0.000	6.38	5.00–8.14	0.000	1.94	1.50–2.52	0.000	3.13	2.42–4.04	*p* < 0.001
	70+	4.51	3.46–5.86	0.000	7.59	5.88–9.79	0.000	2.15	1.64–2.82	0.000	3.55	2.73–4.63	*p* < 0.001
Schooling	Incomplete Elementary	3.95	3.36–4.65	0.000	-	-	-	3.83	3.26–4.51	0.000	-	-	-
	Incomplete Secondary	1.74	1.41–2.14	0.000	-	-	-	1.67	1.35–2.06	0.000	-	-	-
	Complete Secondary	1.00	-	-	-	-	-	1.00	-	-	-	-	-
Region	North	1.73	1.40–2.13	0.000	1.42	1.15–1.76	0.001	1.87	1.51–2.31	0.000	1.51	1.22–1.88	*p* < 0.001
	Northeast	1.82	1.55–2.13	0.000	1.56	1.33–1.84	0.000	1.90	1.62–2.22	0.000	1.60	1.36–1.87	*p* < 0.001
	Southeast	1.00	-	-	1.00	-	-	1.00	-	-	1.00	-	-
	South	1.18	0.97–1.42	0.100	1.26	1.04–1,52	0.018	1.08	0.90–1.31	0.412	1.14	0.95–1.38	0.162
	Center-West	1.25	1.04–1.50	0.018	1.19	0.99–1.43	0.061	1.26	1.05–1.52	0.013	1.18	0.98–1.42	0.081
SE class	D/E	-	-	-	5.14	4.18–6.33	0.000	-	-	-	5.30	4.32–6.49	*p* < 0.001
	C	-	-	-	2.97	2.44–3.62	0.000	-	-	-	2.93	2.41–3.57	*p* < 0.001
	A/B	-	-	-	1.00	-	-	-	-	-	1.00	-	-
At least one NCD	Yes	-	-	-	-	-	-	5.08	4.29–6.01	0.000	5.27	4.45–6.23	*p* < 0.001
	No	-	-	-	-	-	-	1.00	-	-	1.00	-	-

^a^Adjusted for age, sex, and schooling

^b^Adjusted for age, sex, and socioeconomic (SE) class

^c^Adjusted for age, sex, schooling, and diagnosis of at least one non-communicable chronic disease (NCD)

^d^Adjusted for age, sex, socioeconomic class, and diagnosis of at least one non-communicable chronic disease (NCD)

### Healthy life expectancy estimates

The estimated life expectancies, years lived in poor health, and healthy life expectancies by sex and regions, at 20, 40 and 60 years of age are shown in Table 4. Regional differences in healthy life expectancy are higher than the differences in life expectancy for all ages. At twenty years old, HLE estimates in the South and Southeast are 6.2 years higher than in the North and Northeast, for both women and men. At 40 years, the gap decreases to 5.3 years, approximately, and at 60, to 3.5 years.

**Table 4:** Life expectancy (LE), healthy life expectancy (HLE), and lost healthy years (LHY) by age, sex, and Brazilian region, 2013

Region	M			F			T		
	LE	HLE	LHY	LE	HLE	LHY	LE	HLE	LHY
At 20 years of age									
North	50.9	46.3	4.6	57.3	50.9	6.4	53.9	48.5	5.4
Northeast	50.7	46.3	4.4	58.4	51.3	7.1	54.5	48.7	5.8
Southeast	54.8	52.5	2.4	61.1	57.2	3.9	58.0	54.9	3.2
South	55.0	52.1	2.9	61.4	56.1	5.4	58.2	54.1	4.2
Center-West	53.4	49.7	3.6	59.6	54.6	4.9	56.4	52.1	4.3
Brazil	53.3	49.5	3.8	60.1	54.9	5.2	56.7	52.4	4.2
At 40 years of age									
North	34.3	29.7	4.6	38.6	32.8	5.9	36.3	31.2	5.1
Northeast	34.1	30.0	4.1	39.5	33.0	6.5	36.9	31.5	5.3
Southeast	36.7	34.6	2.1	41.8	38.3	3.5	39.3	36.5	2.8
South	36.9	34.0	2.9	42.1	37.2	4.9	39.6	35.6	3.9
Center-West	53.4	49.7	3.6	40.5	35.9	4.6	38.2	34.1	4.1
Brazil	35.8	32.3	3.5	41.0	36.3	4.7	38.5	34.6	3.9
At 60 years of age									
North	18.7	15.3	3.4	21.6	18.0	3.6	20.1	16.7	3.4
Northeast	18.9	16.1	2.7	22.4	17.9	4.4	20.7	17.0	3.7
Southeast	20.4	18.8	1.7	24.1	21.6	2.4	22.4	20.3	2.1
South	20.4	18.2	2.2	24.2	20.6	3.5	22.4	19.5	2.9
Center-West	19.8	16.9	2.9	22.8	19.6	3.3	21.3	18.2	3.1
Brazil	19.9	17.3	2.5	23.4	20.2	3.2	21.7	18.9	2.8

Sources: Life expectancy provided by IBGE [31]; HLE derived from Sullivan’s method using poor self-assessment of health to establish unhealthy state based on PNS data

*M* male, *F* female, *T* total

## Discussion

This is the first study to show inequalities in healthy life expectancy in Brazil by geographic region. Overall, the findings were consistent with previous studies: although women live longer than men, they live relatively fewer years in good health and the percentage of years of healthy life lost increases with age [27, 35]. As to estimates by geographic region, beyond the variation in life expectancy at birth, marked regional inequalities were found in healthy longevity between the least and most developed macro regions of Brazil.

To calculate HLE, we used Sullivan’s method and adopted poor self-rated health as the definition for the unhealthy state. Firstly, because a broader definition of health transcends the absence of death, disease and disability and incorporates concepts of well-being and quality of life [36]. Different from a biomedical assessment of health status, which identifies disease by a set of signs, symptoms and laboratory data, self-perception of health is subjective, combining physical and emotional components of well-being [37]. Secondly, in the context of comparing HLE regional estimates, unhealthy state definitions based on diagnosed morbidity do not work well as they depend on access to diagnosis, admittedly uneven by region and area of residence (urban/rural) [9, 38]. A limitation of this study, however, is that data on functional limitations of daily activities are not available for the total PNS sample, only for persons aged 60 years and over, and so could not be used as an estimator of health status.

With the current growth in longevity experienced by populations throughout the world, the proportion of years not lived in full health will likely increase, and measures of unhealthy state are becoming increasingly important. Different techniques have been proposed to refine the simple binary measures of health states, like quality-adjusted life years [39] or the Canadian composite index of wellbeing based on eight domains of health [40]. Nevertheless, the recurrent utilization of self-rated health comes from its validity, established by its association with more objective measures of health disorders [23] and its potential use to identify already disabled persons [41].

A previous study in Brazil based on the World Health Survey (WHS), 2003 compared three measures of unhealthy states: poor self-rated health; presence of a long-term disease or disability that limits daily activities; and a principal component score based on severity of functional limitations resulting or not from long-term illness. Although more complex, the two approaches that included presence of functional limitations produced very similar results to the estimate based on self-rated health, especially in the elderly [27].

As the WHS sample does not allow us to make statistical inferences by geographic region [42], we compared the PNS (2013) and WHS (2003) results at the national level. Life expectancy at 20 years old increased more than 2 years and at 60 years old, more than one year. However, the largest increase was found in healthy life expectancy. Ten years later, considering exactly the same question for self-rated health, the number of lost healthy years decreased from 6.9 to 4.2 at 20 years old, and from 4.8 to 2.8 at 60 years old. As has been shown before, Brazilians are living longer and feeling better [25].

Given the significant growth of non-communicable chronic diseases and disabling disorders in the Brazilian population [19], the HLE increase is apparently paradoxical. A likely explanation is the improvement of quality of life of the Brazilian population in terms of socioeconomic conditions and access to health care [29, 43, 44], in the so called virtuous circle generated by the large social investments in the country. Recent national studies have also shown the influence of better-quality life conditions, expansion of primary health care, and the impact of the income inequality reduction on morbidity and mortality indicators [45–47].

In respect to regional variation, our results showed pronounced inequalities in life expectancy, confirming previous mortality studies [48]. Significant regional differences in poor self-rated health were also found. Comparative statistical analysis between the North and Northeast regions to the Southeast showed significant effects, even after controlling for age, sex, diagnosis of at least one non-communicable chronic disease, and schooling or socioeconomic class. These results indicate that regional differences in health self-perception reflect socio-structural influences, which go beyond the concentration of poverty and educational attainment [49], as well as factors that determine different experiences of illness as social consequences of living in disadvantaged areas [37].

As to regional inequality in healthy years, the results suggest that HLE is a more sensitive indicator than life expectancy. At age 60, the loss of healthy years is twice as high in the North as in the Southeast. These findings are in accordance with previous national and international studies. A study in the city of Rio de Janeiro showed huge HLE differences in the elderly, by comparing socioeconomic strata within the city [50]. Similar analyses in South Korea showed the importance of considering community-level socioeconomic conditions as key correlates of survival [49]. In the United States, large HLE inequalities have also been reported by geographic region [51].

Evidence of the effects of socioeconomic inequalities on healthy longevity are increasingly available, as well. Results are invariably unfavourable to the disadvantaged groups [52–54]. In England, a recent study combined survey data on health-related quality of life with mortality data and found a difference of approximately 11 lost years of quality of life between the most and least healthy quintile groups [55]. A study in European countries showed large and increasing inequalities in healthy life expectancy at age 50 from 2005 to 2010, partly explained by worsening of material deprivation and long-term unemployment [56].

By showing that not only mortality indicators are associated with living conditions, but that regional inequalities are even more pronounced when well-being is taken into consideration, this study renews attention to the need for promoting actions and programs to decrease socio-spatial gaps. The inclusion of health self-assessment in addition to the mortality component in a single health indicator demonstrated the excess burden of poor health experienced by populations in the less developed regions. To mitigate the effects of social exclusion, the development of strategies at the regional level is essential not only to provide health care to all persons in need, but also to reduce risk exposures and to support prevention policies for adoption of healthy behaviors, prioritizing the disadvantaged population groups that will have the greater impact of interventions.

## Conclusions

The findings suggest that wellness should be taken into account while monitoring geographic health inequalities, especially among elderly, and point to the adoption of HLE as an outcome measure at the national, regional, state, and community levels to compare the effectiveness of health interventions and evaluate disparities. In this context, the results presented here can be used as a baseline to monitor targets in healthy life expectancy and in regional health inequalities.

## Abbreviations

PNS: National health survey
HLE: Healthy life expectancies
MoH: Ministry of health
IBGE: Brazilian Institute of Geography and Statistics
CONEP: National Commission of Ethics in Research
SIPD: Master sample of the integrated household survey system
PSU: Primary sampling unit
CNEFE: National register of addresses for statistical purposes
PDA: Personal digital assistants
OR: Odds-ratio
WHS: World Health Survey
